# Mathematical modeling identifies optimum lapatinib dosing schedules for the treatment of glioblastoma patients

**DOI:** 10.1371/journal.pcbi.1005924

**Published:** 2018-01-02

**Authors:** Shayna Stein, Rui Zhao, Hiroshi Haeno, Igor Vivanco, Franziska Michor

**Affiliations:** 1 Department of Biostatistics, Harvard T. H. Chan School of Public Health, Boston, Massasschusetts, United States of America; 2 Department of Biostatistics and Computational Biology, Dana-Farber Cancer Institute, Boston, Massachusetts, United States of America; 3 Department of Stem Cell and Regenerative Biology, Harvard University, Cambridge, Massachusetts, United States of America; 4 The Broad Institute of Harvard and MIT, Cambridge, Massachusetts, United States of America; 5 Department of Biology, Kyushu University, Fukuoka, Japan; 6 Division of Cancer Therapeutics, The Institute of Cancer Research, London, United Kingdom; 7 Center for Cancer Evolution, Dana-Farber Cancer Institute, Boston, Massachusetts, United States of America; H. Lee Moffitt Cancer Center and Research Institute, UNITED STATES

## Abstract

Human primary glioblastomas (GBM) often harbor mutations within the epidermal growth factor receptor (EGFR). Treatment of EGFR-mutant GBM cell lines with the EGFR/HER2 tyrosine kinase inhibitor lapatinib can effectively induce cell death in these models. However, EGFR inhibitors have shown little efficacy in the clinic, partly because of inappropriate dosing. Here, we developed a computational approach to model the *in vitro* cellular dynamics of the EGFR-mutant cell line SF268 in response to different lapatinib concentrations and dosing schedules. We then used this approach to identify an effective treatment strategy within the clinical toxicity limits of lapatinib, and developed a partial differential equation modeling approach to study the *in vivo* GBM treatment response by taking into account the heterogeneous and diffusive nature of the disease. Despite the inability of lapatinib to induce tumor regressions with a continuous daily schedule, our modeling approach consistently predicts that continuous dosing remains the best clinically feasible strategy for slowing down tumor growth and lowering overall tumor burden, compared to pulsatile schedules currently known to be tolerated, even when considering drug resistance, reduced lapatinib tumor concentrations due to the blood brain barrier, and the phenotypic switch from proliferative to migratory cell phenotypes that occurs in hypoxic microenvironments. Our mathematical modeling and statistical analysis platform provides a rational method for comparing treatment schedules in search for optimal dosing strategies for glioblastoma and other cancer types.

## Author summary

*In vivo* inhibition of tumor expansion requires a sufficient amount of therapeutic agent to be present in the tumor tissue. A number of factors affect drug concentrations including the maximum tolerated dose, pharmacokinetics and pharmacodynamics profiles. We present a computational modeling platform incorporating both in vitro data and published clinical trial data to investigate the efficacy of lapatinib as a function of different dosing schedules for inhibiting glioblastoma tumor cell growth. The goal of our method is to find the best dosing schedule balancing both toxicity and efficacy. Our modeling approach identifies continuous dosing as the best clinically feasible strategy for slowing down tumor growth even when taking into consideration intratumor heterogeneity, drug resistance and reduced lapatinib concentrations in the tumor due to the blood brain barrier.

## Introduction

Glioblastoma is the most common and aggressive form of brain tumors in adults, characterized by short survival and poor treatment response [1]. Currently, the standard of care for glioblastoma patients includes surgery followed by radiotherapy and adjuvant chemotherapy with temozolomide [2]. However, the addition of chemotherapy only modestly prolongs survival (median 14.6 months) compared to radiation alone (median 12.1 months). Thus, there remains a pressing unmet medical need for more effective therapeutic agents. Unfortunately, since the introduction of temozolomide, no other compound has been able to significantly prolong patient survival in clinical trials. For orally administered drugs, most trials have only explored daily continuous dosing schedules (Table 1). However, there is increasing evidence that for some targeted agents, intermittent schedules can deliver equal or potentially even superior therapeutic benefit with less toxicity [3, 4].

**Table 1:** Dosing strategies for orally administrated drugs for GBM from published clinical trials.

Study	Treatment	Patients (N)	Dosing strategies
Rich et al. [77]	Gefinitib	53	500-1000mg daily
Prados et al. [78]	Erlotinib	83	100-500mg daily
Brown et al. [79]	Erlotinib	100	150mg daily
Reardon et al. [80]	Imatinib	33	500mg twice daily
Wen et al. [81]	Imatinib	55	600-800mg daily
Raymond et al. [82]	Imatinib	112	600mg daily—500mg twice daily
Reardon et al. [15]	Lapatinib	41	1000mg twice daily
Thiessen et al. [16]	Lapatinib	17	1000-1500mg twice daily
Chien et al. [17]	Lapatinib	25	1000-5250mg twice daily for two days in a 5-day cycle

In the last decade, several molecularly targeted agents that inhibit recurrently mutated proteins have been investigated as a therapeutic strategy in glioblastoma. These have included several inhibitors of the epidermal growth factor receptor (EFGR), which is mutationally activated in approximately 50% of adult GBMs. Lapatinib is a small molecule tyrosine kinase inhibitor of human epidermal receptor 2 (HER2) and EGFR, which currently has regulatory approval for the treatment of HER2-positive advanced or metastatic breast cancer [5]. Additional indications for lapatinib [6–14] including glioblastoma, are currently being explored [15, 16]. In GBM, clinical trials of lapatinib have failed to show efficacy using continuous dosing [15, 16]. Interestingly, a study that evaluated EGFR inhibition as a means to prime tumor vasculature for efficient delivery of chemotherapy showed that in glioblastoma patients, a 2-day pulse of high dose (5,250 mg/day) lapatinib given through twice daily dosing was well tolerated [17]. However, the question remains whether an altered dosing strategy might increase the efficacy of this agent. Due to ethical concerns of testing several dosing strategies in the absence of preclinical data suggesting their benefit, as well as to speed up discovery, mathematical modeling of treatment response can be used to identify predicted best administration schedules. Here, we explored continuous and pulsatile dosing strategies in an EGFR-mutant GBM cell line, and used mathematical and statistical modeling to determine optimal lapatinib dosing schedules for inhibiting tumor growth.

The use of mathematical models for treatment optimization is part of a growing effort to improve clinical trial design for cancer patients. Many models have been developed to investigate the relationship between toxicity and dose in an attempt to better characterize the toxicity profile of the therapeutic products under investigation. For instance, Huitema et al. reviewed several differential equation-based models of chemotherapy-induced myelosuppression, cardiovascular events and other ordinal adverse events [18]. Amantea et al. developed a model incorporating exposure, biomarkers, efficacy endpoints and adverse events in models describing the treatment response of patients with gastrointestinal stromal tumors [19]. Their analysis uncovered a correlation between adverse events and efficacy: drug-induced increases in diastolic blood pressure were positively associated with overall survival. Another study by Huitema et al. examined the effects of the anti-angiogenic drug E7080 and its drug-induced adverse events in an attempt to evaluate dosing regimens with regard to their reduction of adverse events and improvement of efficacy [20]. They found that proteinuria could be best described by a discrete-time Markov transition model. Similarly, Fuhr et al. constructed a continuous-time Markov model to investigate erlotinib-induced adverse events in non-small cell lung cancer patients; their simulation results provided support for the use of high-dose pulses as an alternative dosing strategy for addressing acquired resistance often found when using a low continuous dose [21]. In these examples, a delicate balance between toxicity and efficacy determines the most efficacious dose.

In addition to understanding toxicity profiles, mathematical models have also been used to model pre-clinical data from animal or in vitro systems in order to bypass the difficulty associated with directly observing tumor progression in humans. Translational models incorporating in vitro or animal data have also be developed to investigate the relationship between efficacy endpoints and exposure. Mould et al. provided a comprehensive review of how the use of mathematical models can aid early development of anti-cancer therapy, in particular for describing tumor volume as a function of drug exposure [22]. Most papers these authors discussed describe changing tumor volumes using differential equation models with two terms: net tumor growth in the absence of therapeutic candidates and a drug-induced shrinkage effect [23–27]. These models suggest that the best strategy for inhibiting tumor growth is to maximize the effect of drug exposure, which often corresponds to higher drug concentrations. However, given toxicity constraints, high drug concentrations usually cannot be achieved for long time periods. An alternative strategy is to administer drugs in pulsatile doses in order to circumvent the toxicity limit. A small trial using weekly pulsatile high-dose erlotinib showed promising results for controlling central nervous system metastases from epidermal growth factor receptor-mutant non-small cell lung cancer [28]. Crooke et al. also performed computational analyses comparing continuous and pulsatile dosing and found that pulsed therapy is more effective than continuous therapy [29]. A clinical trial [30] based on mathematical modeling and preclinical [31] experiments demonstrated that a combined low-dose continuous and high-dose pulsed erlotinib schedule was successful at preventing progression in patients with CNS metastases but did not show significantly delayed emergence of resistance due to the T790M EGFR mutation.

**Fig 1:**
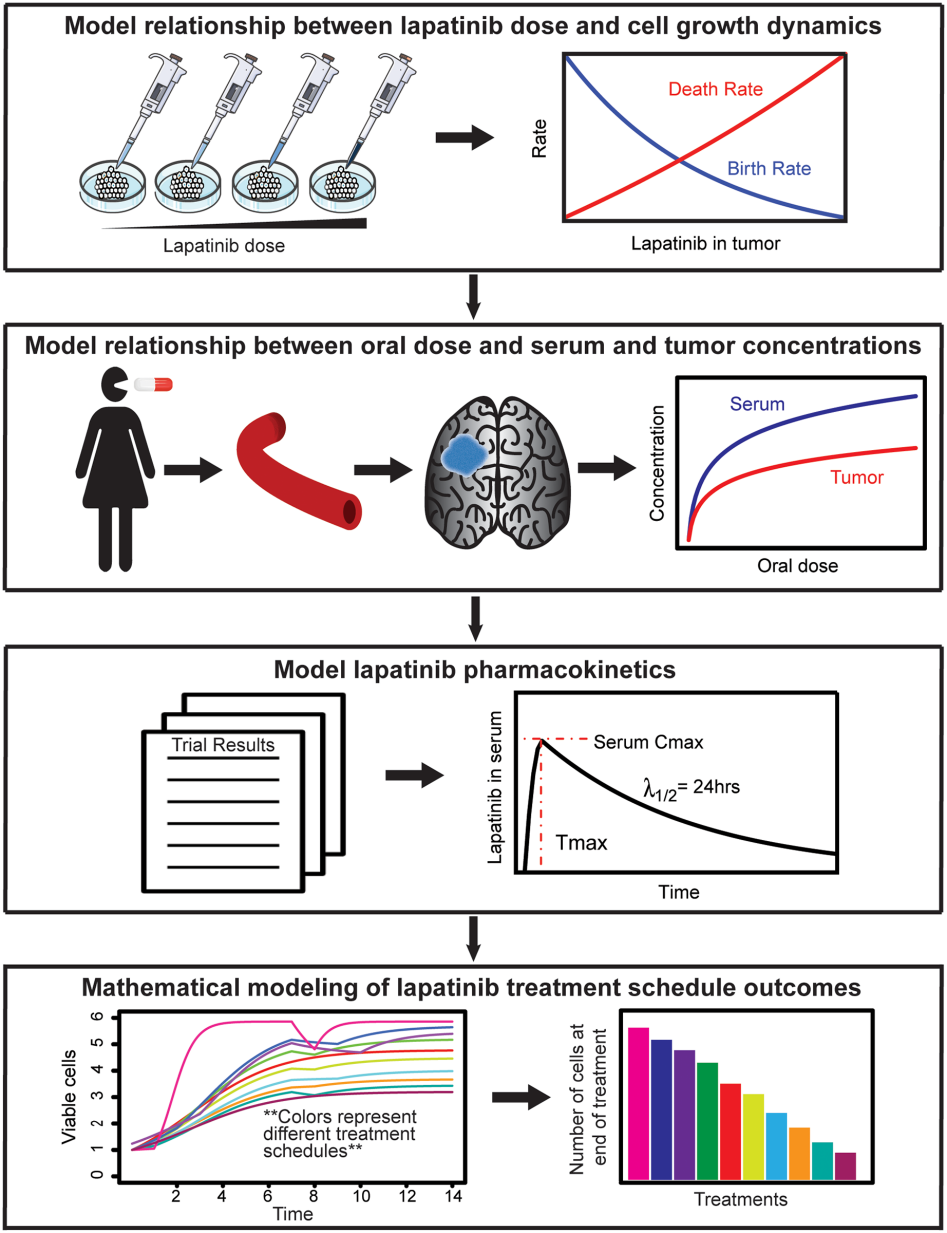
Illustration of the combined experimental and computational modeling approach. First, we establish the relationship between tumor lapatinib concentration and tumor cell dynamics; second, we identify the relationship between oral lapatinib dose, serum lapatinib concentration, and tumor lapatinib concentration; third, we ascertain the pharmacokinetics model describing lapatinib uptake and decay; lastly, we build a mathematical model to compare the efficacies of different lapatinib treatment schedules.

Here we present a computational modeling approach incorporating both toxicity data from phase I clinical trials and efficacy data from pre-clinical research to examine the effects of various lapatinib dosing schedules on tumor growth (Fig 1). The goal of our work is to determine the best dosing strategy given the observed toxicity limit. Specifically, we are interested in answering the question whether alternative dosing strategies can be applied to circumvent the toxicity limit while exceeding the level of efficacy observed in continuous dosing of lapatinib while taking into account known characteristics of GBM growth and treatment response such as diffusivity, intratumor heterogeneity, and the blood brain barrier.

## Materials and methods

### Cell culture

SF268 cells were obtained from the National Cancer Institute (NIH) and routinely grown in DMEM supplemented with 10% fetal bovine serum. To assess the effects of drug treatment, 150,000 cells were seeded on 6-cm dishes and allowed to attach overnight. Cells were then switched to growth media supplemented with 1% fetal bovine serum and the indicated concentrations of lapatinib or DMSO (vehicle). Each treatment condition was done in triplicate. For treatment discontinuation, cells were washed 3 times with media containing vehicle, and then allowed to continue growth in media containing vehicle. Vehicle-treated cells were also washed 3 times to control for the effects of washing. Cell viability and cell death were evaluated by the trypan blue exclusion assay using a ViCell cell viability analyzer (Beckman Coulter).

### Mathematical modeling

#### *In vitro* cell dynamics

To describe *in vitro* cell dynamics, we designed a differential equation model of cell growth given by

$$\begin{array}{c} \frac{dY_{1}}{dt}=bY_{1}\left(1-\frac{Y_{1}}{K}\right)-dY_{1} \end{array}$$ (1a)

$$\begin{array}{c} \frac{dY_{2}}{dt}=dY_{1}-cY_{2} \end{array}$$ (1b)

where *Y*_1_ and *Y*_2_ denote the numbers of viable and dead cells, respectively; the parameters *b* and *d* denote the birth and death rates of viable cells; *K* denotes the carrying capacity of the *in vitro* assay system; and *c* denotes the clearance rate of dead cells.

#### Grid search algorithm for estimating model parameters

We implemented a grid search algorithm, given by

$$\begin{array}{c} \underset{b,c,d}{argmin}\left\{{\left(Y_{1,obs}\left(t\right)-Y_{1}\left(b,d,c,t\right)\right)}^{2}+{\left(Y_{2,obs}\left(t\right)-Y_{2}\left(b,d,c,t\right)\right)}^{2}\right\} \end{array}$$ (2)

which minimizes the squared distance between the observed and predicted cell numbers for each experimental setting in order to estimate model parameters that best recapitulate the experimental results.

#### Characterizing the relationship between birth and death rates and lapatinib dose

We used an exponential model to describe the relationship between birth and death rates and lapatinib dose given by

$$\begin{array}{c} b\left(L\right)=b_{0}e^{b_{1}L}, \end{array}$$ (3a)

$$\begin{array}{c} d\left(L\right)=d_{0}e^{d_{1}L}, \end{array}$$ (3b)

where *b*_0_ and *b*_1_ are the intercept and slope parameters, respectively, describing the birth rate, and *d*_0_ and *d*_1_ are the intercept and slope parameters, respectively, describing the death rate.

#### Patient-derived toxicity constraints

To characterize the relationship between the maximally tolerated lapatinib dose per treatment and number of treatment days, we fit a linear model given by

$$\begin{array}{c} y_{dose}=\alpha_{0}+\alpha_{1}x_{days} \end{array}$$ (4)

and an exponential model given by

$$\begin{array}{c} y_{dose}=e^{\beta_{0}+\beta_{1}x_{days}}, \end{array}$$ (5)

where *y*_*dose*_ denotes the lapatinib intake per treatment day and *x*_*days*_ is the number of treatment days per treatment cycle.

#### Lapatinib pharmacokinetics

We fit a logarithmic function to model lapatinib absorption into the blood stream, according to

$$\begin{array}{c} y_{serum}\left(ng/mL\right)=\tau_{0}+\tau_{1}log\left(x_{oral}\left(mg\right)\right), \end{array}$$ (6)

where *x*_*oral*_ is the dose of oral lapatinib in miligrams and *y*_*serum*_ is the concentration of lapatinib in the serum in ng/mL units.

To convert serum to tumor lapatinib concentration, we used the conversion

$$\begin{array}{c} y_{tumor}\left(nM\right)=0.61\times y_{serum}\left(ng/mL\right)\times \frac{mol}{943.5\times 10^{9}ng}\times \frac{1000mL}{1L} \end{array}$$ (7)

where *y*_*serum*_ is the serum concentration in ng/mL, *y*_*tumor*_ is the concentration of lapatinib in the tumor, and 0.61 was the mean tumor/serum ratio reported in Vivanco et al. [32].

#### Modeling *in vivo* GBM growth using a diffusion equation

We used the following diffusion model to simulate *in vivo* GBM growth

$$\begin{array}{c} \frac{\partial c}{\partial t}=D\nabla^{2}c+\rho c\left(1-\frac{c}{\kappa }\right), \end{array}$$ (8)

where *c*(*x*, *t*) is the concentration of cells in 1*mm*^3^ volume of tissue at position *x* and time *t*, *D* is the diffusion coefficient defining the net rate of migration of tumor cells, *ρ* is the net proliferation rate of cells per day, *κ* is the carrying capacity of the tissue, and ∇^2^ is the Laplacian.

To model GBM growth during treatment, we scale *ρ* by the birth and death rates estimated from the *in vitro* lapatinib treatment data such that

$$\begin{array}{c} \rho \left(L\left(t\right)\right)=\rho_{0}\frac{b(L(t))-d(L(t))}{b(0)-d(0)}, \end{array}$$ (9)

where *ρ*_0_ = 0.0387 is the proliferation rate in the absence of treatment [33], *L*(*t*) is the concentration of lapatinib in the tumor at time *t*, *b*(0) and *d*(0) are the birth and death rates in the absence of treatment, and *b*(*L*(*t*)) and *d*(*L*(*t*)) are the birth and death rates during treatment with *L*(*t*) concentration of lapatinib. The diffusion model during treatment is then given by

$$\begin{array}{c} \frac{\partial c}{\partial t}=D\nabla^{2}c+\rho \left(L\left(t\right)\right)c\left(1-\frac{c}{\kappa }\right). \end{array}$$ (10)

Lastly, we assumed an isolated system corresponding to a no-flux boundary condition of the form

$$\begin{array}{c} \frac{\partial c(0,t)}{\partial x}=\frac{\partial c(R,t)}{\partial x}=0,\;0\leq t\leq T \end{array}$$ (11)

where *T* is the simulation time and R is the radius of the computational domain.

#### Modeling GBM heterogeneity using diffusion equations

We used the following system of diffusion equations to model intratumor heterogeneity due to the presence of drug-sensitive and -resistant cells:

$$\begin{array}{c} \frac{\partial c_{S}}{\partial t}=D\nabla^{2}c_{S}+\rho \left(L\left(t\right)\right)c_{S}\left(1-\frac{c_{R}+c_{S}}{\kappa }\right), \end{array}$$ (12a)

$$\begin{array}{c} \frac{\partial c_{R}}{\partial t}=D\nabla^{2}c_{R}+\rho_{0}c_{R}\left(1-\frac{c_{R}+c_{S}}{\kappa }\right), \end{array}$$ (12b)

where *c*_*S*_ and *c*_*R*_ represent the concentration of sensitive and resistant cells at position *x* and time *t*. The proliferation rate in Eq (12b) given by *ρ*_0_, the proliferation rate in the absence of treatment, because the resistant cells are assumed to be unaffected by treatment.

#### Modeling reduced lapatinib delivery to the brain

To model reduced lapatinib delivery to the brain due to the blood brain barrier or other reasons, we used a modified diffusion model given by

$$\begin{array}{c} \frac{\partial c}{\partial t}=D\nabla^{2}c+\rho \left(Q*L\left(t\right)\right)c\left(1-\frac{c}{\kappa }\right), \end{array}$$ (13)

where *Q* denotes the percent of the serum concentration of lapatinib that reaches the tumor.

#### Modeling the Go-or-Grow mechanism

It is well known that glioblastoma is highly hypoxic, and that one mechanism used by tumor cells to survive in hypoxic environments is a phenotypic switch from proliferative to migratory phenotypes [34], also known as the “Go-or-Grow” mechanism. This phenotypic switch depends on the oxygen concentration in the microenvironment, which we modeled using the following system of three PDEs describing migratory cell, proliferative cell, and oxygen concentration, repectively [35]:

$$\begin{array}{c} \frac{\partial c_{M}}{\partial t}=D\nabla^{2}c_{M}-f_{MP}\left(\sigma \right)c_{M}+f_{PM}\left(\sigma \right)c_{P}, \end{array}$$ (14a)

$$\begin{array}{c} \frac{\partial c_{P}}{\partial t}=\rho \left(L\left(t\right)\right)c_{P}\left(1-\frac{c_{M}+c_{P}}{\kappa }\right)+f_{MP}\left(\sigma \right)c_{M}-f_{PM}\left(\sigma \right)c_{P}, \end{array}$$ (14b)

where *c*_*M*_(*x*, *t*) and *c*_*P*_(*x*, *t*) represent the concentration of migratory and proliferative tumor cells, respectively, at position *x* and time *t*, and *σ*(*x*, *t*) is the oxygen concentration at position *x* and time *t*. The latter is given by the diffusion equation

$$\begin{array}{c} \frac{\partial \sigma }{\partial t}=D_{\sigma }\nabla^{2}\sigma +h_{1}v\left(\sigma_{0}-\sigma \right)-h_{2}\left(c_{M}+c_{P}\right)\sigma , \end{array}$$ (15)

where *D*_*σ*_ is the oxygen diffusion coefficient, *h*_1_ is the transvasculature permeability, *h*_2_ is the rate at which glioma cells consume oxygen, and *σ*_0_ is the oxygen concentration in normal brain cells. Lastly, *f*_*MP*_(*σ*) and *f*_*PM*_(*σ*) represent the rates at which tumor cells switch from migratory to proliferative and proliferative to migratory phenotypes, respectively. Assuming the rates of switching have a linear dependence on oxygen concentration, the two functions are then given by

$$f_{MP}\left(\sigma \right)=\lambda_{2}\sigma ,$$ (16a)

$$f_{PM}\left(\sigma \right)=\lambda_{1}-\sigma ,$$ (16b)

where λ_1_ and λ_2_ are positive constants.

#### Testing the relationship between cell motility and treatment response

To test whether the relationship between the cell motility predicted by the diffusion equation and the tumor volume depends on the treatment schedule, we used a linear regression model with interaction terms:

$$\begin{array}{c} V_{S}=\nu_{0}+\nu_{D}D+\nu_{S}S+\nu_{int,S}D*S=\left(\nu_{0}+\nu_{S}*S\right)+\left(\nu_{D}+\nu_{int,S}S\right)D. \end{array}$$ (17)

Here *D* is the diffusion parameter, *S* is a categorical variable representing the treatment schedule used, and *V*_*S*_ is the volume at the end of treatment with treatment schedule *S*. Then, (*ν*_*D*_ + *ν*_*int*, *S*_ *S*) is the slope representing the effect of motility on the tumor volume at the end of treatment, and *ν*_*int*, *S*_ *S* is the effect of the interaction between cell motility and the treatment schedule. We used schedule 1 as the baseline schedule, meaning that *ν*_*S*_ = *ν*_*int*, *S*_ = 0. Thus we can test *H*_0_: *ν*_*int*, *S*_ = 0 to assess whether tumor growth under schedule *S* is more or less affected by increased cell motility than schedule 1, for *S* = schedule 2, schedule 3, schedule 4, or schedule 5.

#### Simulating a clinical trial of treatment schedules

A cohort of *N* = 50 patients was used to simulate each clinical trial to test for differences between treatment schedules. Cell dynamics and pharmacokinetics parameters were randomly sampled for each patient. The same 50 patients were used to evaluate survival outcomes for different treatment schedules. Cell birth and death rate parameters, *b*_0_, *b*_1_, *d*_0_, and *d*_1_, were sampled from $Normal(\hat{\mu },\hat{\sigma })$ distributions, where $\hat{\mu }$, and $\hat{\sigma }$ are the estimated parameters and corresponding standard errors listed in Table 2. Lapatinib concentration absorption from the serum into the tumor was sampled from a *Beta*(2.3, 1.5) distribution, which has mean 0.61, the average tumor/serum concentration reported in [32].

**Table 2:** Estimated pharmacodynamic and pharmacokinetic parameters.

Parameter	Estimate	Standard Error	p-value
*b*_0_	1.4153	0.115	9.286 × 10^−8^
*b*_1_	-0.001043	1.392 × 10^−4^	1.204 × 10^−5^
*d*_0_	0.02159	0.0055	0.00237
*d*_1_	8.418 × 10^−4^	9.816 × 10^−5^	3.351 × 10^−6^
*α*_0_	9.02464	0.06392	0.00451
*α*_1_	-0.20599	0.02021	0.06227
*β*_0_	7596.2	632.0	0.0528
*β*_1_	-942.3	199.9	0.1330
*τ*_0_	-2.1987	1.1334	0.0843
*τ*_1_	0.4959	0.1541	0.0105

*In silico* trials that additionally varied cell motility sampled the diffusion parameter from a *Gamma*(1, 1) distribution that was truncated below 0.55 and above 4.23, the diffusion parameter thresholds reported in Murray, 2012 [36]. Diffusion parameter values were then divided by 30 to convert from mm^2^/month to mm^2^/day.

## Results

### *In vitro* tumor cell pharmacodynamics

**Fig 2:**
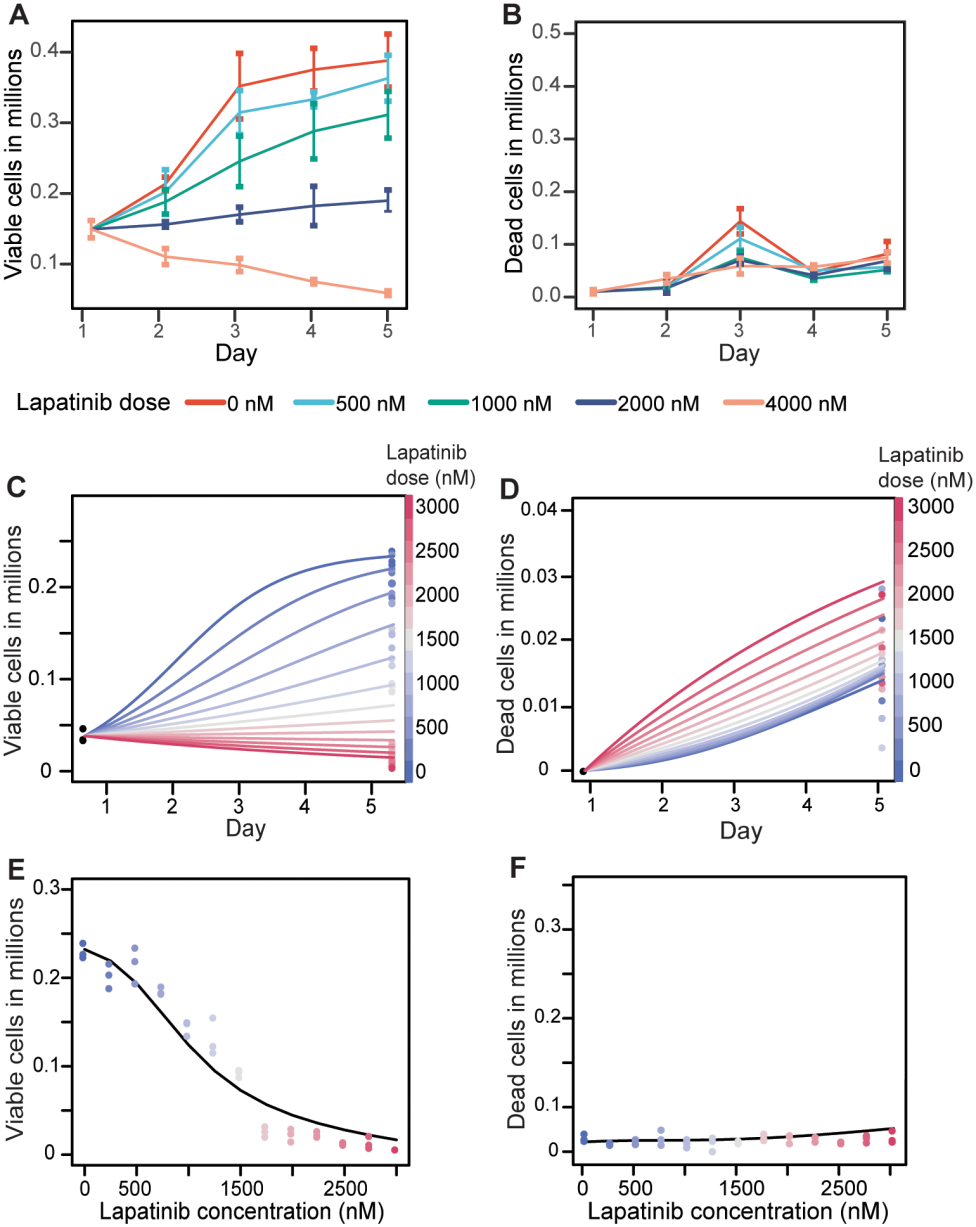
Observed and model-predicted numbers of viable and dead cells over time for varying concentrations of lapatinib. A: The observed number of viable cells over a 5 day period in the presence of varying lapatinib concentrations. B: The observed number of dead cells over a 5 day period in the presence of varying lapatinib concentrations. C: The viable cell trajectories for different concentrations of lapatinib based on the logistic ODE model, where different colored lines represent the model-predicted viable cell growth trajectories for different lapatinib concentrations. D: The dead cell trajectories for different concentrations of lapatinib based on the logistic ODE model, assuming a clearance rate of zero. E-F: Comparisons between model-predicted and observed numbers of viable and dead cells.

We first modeled the *in vitro* cell pharmacodynamics during lapatinib treatment using a logistic ordinary differential equation (ODE) model (Eq 1). The choice of a logistic model over simpler exponential models was made based on exploratory analysis of viable cell dynamics over time (Fig 2A and 2B). During treatment with low concentrations of lapatinib, the growth rate of cells leveled off after 5 days; this change in the growth rate as a function of the cell number is better captured by a logistic than an exponential model. Note that the birth, death, and clearance rates and carrying capacity are restricted to be non-negative real numbers. The carrying capacity, *K*, is assumed to be invariant to lapatinib concentrations and is estimated as the maximum observed number of viable cells on day 5 in the absence of lapatinib. The birth, death, and clearance rate parameters *b*, *d*, and *c*, respectively, are functions of the lapatinib concentration; however, the exact functional form describing the relationship between these rates and the lapatinib dose is unknown.

To estimate the birth, death, and clearance rates that best capture cell growth patterns, we implemented a grid search algorithm (Eq 2) to minimize the squared distance between observed and predicted cell numbers for each experimental setting in order to determine the parameters that best reproduce observed cell numbers. The patterns of birth, death, and clearance rates against lapatinib concentration are shown in S1A–S1C Fig. We observed that, when allowing the clearance rates to vary for different concentrations of lapatinib, only the death rates show a discernible pattern with increasing lapatinib dose (S1B Fig). However, if the clearance rate is constrained to a constant, we observed that both birth and death rates demonstrated clear dose-dependent correlations (S1D–S1F Fig). We thus adopted the latter approach, leading to birth rates exponentially decreasing and death rates exponentially increasing with escalating lapatinib concentrations. To further investigate the effects of a constant clearance rate, we studied a wide range of values for the clearance rate (0.0, 0.1, 0.2, 0.3, 0.4, and 0.5), and found only minor differences in the birth and death rates estimated for different clearance rates. We therefore selected the simplest assumption with a clearance rate of 0, which reduces the final model to Eq (1), with *c* = 0 in Eq (1b).

We then modeled the relationship between birth and death rates and lapatinib dose as exponential distributions (Eq 3). We estimated the distribution coefficients for birth and death rates using nonlinear least squares regression, which are shown in Table 2. We found that birth and death rates are both significantly associated with lapatinib dose (*p*_*birth*_ = 1.204 × 10^−5^, *p*_*death*_ = 3.351 × 10^−6^).

Once we established the relationship between the birth and death rates and the lapatinib concentration, we modeled the growth trajectory for different lapatinib concentrations over time (Fig 2C and 2D) using the model described in Eq 1. We found that the number of viable cells predicted by our model agrees with the observed numbers of viable cells on day 5 (Fig 2E). The model also predicts a clear negative correlation between the lapatinib concentration and the number of viable cells at day 5; however, our model agrees less well with the observed number of dead cells on day 5 (Fig 2F). One possible reason for the discrepancy is that the number of observed dead cells in much smaller than the number of observed viable cells, and hence birth and death rates were determined predominantly using the observed viable cells. Note that our approach does not capture the peak in the number of dead cells on day 3; we believe that this peak does not represent true underlying biology but rather represents a technical artifact since, if we were to include the peak in our analysis, we would estimate growth and death rates that are inconsistent with the know action of anti-cancer agents (i.e. increasing doses lead to lower proliferation and/or enhanced death). Designing a model to capture this peak would likely lead to overfitting and hence decreased predictive abilities. For these reasons we decided to use the approach outlined above to describe the cell growth trajectories in the presence of lapatinib treatment.

### Patient-derived toxicity constraints

**Fig 3:**
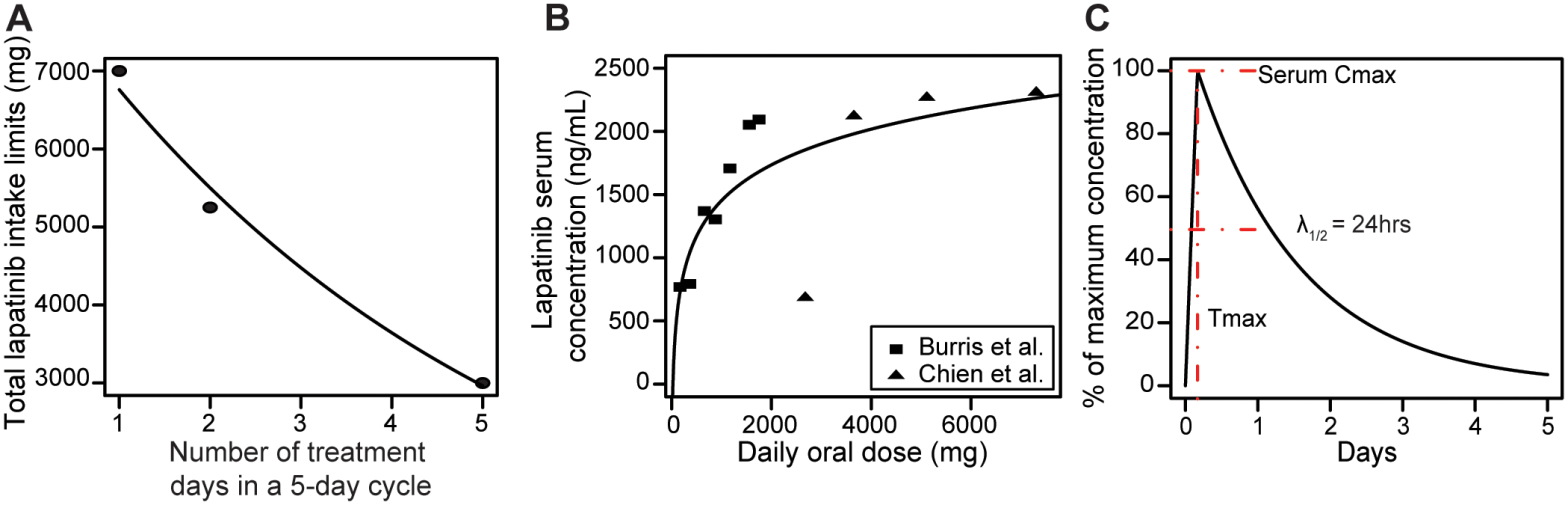
Maximum tolerated dose per treatment cycle and lapatinib pharmacokinetics. A: Maximally tolerated oral lapatinib dose as a function of the numbers of treatment days in a 5-day treatment cycle. The exponential function shown in black is estimated based on the three data points. B: The relationship between oral doses and maximum serum lapatinib concentrations at Cmax, 4 hours after oral intake. A logarithmic function is fitted to estimate the relationship between oral doses and serum concentrations. C: The lapatinib pharmacokinetic profile for modeling serum clearance is determined as a linear-exponential function with *C*_*max*_ = 4 hours and *T*_1/2_ = 24 hours.

To identify optimum treatment schedules for the delivery of lapatinib to patients, our approach needs to take into account clinically determined toxicity constraints—the maximally tolerated dose (MTD) for a particular time interval that does not lead to dose-limiting side effects. The maximum allowable oral dose as a function of the number of treated days was constructed from two clinical studies: Thiessen et al. [16] and Chien et al. [17]; a third point was selected based on clinical expertise in order to estimate the shape of this function (Fig 3A). The three toxicity limits we then fitted to both a linear (Eq 4) and an exponential (Eq 5) model (Table 2). We found that the exponential model better explains the observed limiting toxicity as a function of the number of treatment days (AIC values: linear 49.48 vs. exponential 5.712). Limiting doses were extrapolated based on the exponential model for 1 to 5 treatment days in a 5-day treatment cycle.

### *In vivo* lapatinib pharmacokinetics

In addition to modeling tumor cell dynamics in the presence of lapatinib treatment, our approach also requires a model of lapatinib pharmacokinetics—the dynamics of lapatinib absorption into the blood stream, absorption into the tumor, and clearance from the tumor. We first modeled lapatinib absorption into the blood stream using pharmacokinetic parameters from clinical trials (Table 4 in Burris et al [37] and Table 4B in Chien et al. [17]). We then fit a logarithmic function (Eq 6) to this data to describe the relationship between the limiting oral doses identified above to the maximum plasma concentration (Fig 3B, Table 2).

To convert the plasma concentration to tumor concentration, we used patient pharmacokinetic data reported in Table S4 in Vivanco et al. [32]. In this study, the authors found the mean tumor/serum concentration to be 0.61 in biopsied GBMs following 1 week of daily lapatinib treatment. The resulting serum to tumor conversion function is described in Eq 7.

Finally, we constructed a time-dependent pharmacokinetic model using parameters *C*_*max*_ = 4 hours, i.e. the time to reach the maximum serum concentration after drug administration, and *T*_1/2_ = 24 hours, i.e. the time to reach half of the maximum concentration after *C*_*max*_ [38]. A linear function was used to model lapatinib uptake from 0 to 4 hours, and an exponential function was used to model the subsequent decay pattern, with a half-life of 24 hours (Fig 3C).

### Optimum dosing strategies for lapatinib treatment

Combining tumor cell dynamics with patient derived lapatinib pharmacokinetics provides the *in vivo* tumor growth trajectory—the basis for comparing different dosing strategies. In particular, we compared five treatment schedules that provide the MTD per day according to the identified toxicity constraints, including one continuous dosing strategy and four pulsatile dosing strategies (Table 3).

**Table 3:** Lapatinib concentrations for MTD treatment schedules.

Schedule	Treatment Days	Oral (mg)	Serum (ng/mL)	In tumor (nM)
1	5	3000	1972	1275
2	4	3642	2083	1347
3	3	4475	2202	1424
4	2	5250	2294	1483
5	1	7000	2460	1590

#### Optimum dosing strategies using a logistic ODE growth model

**Fig 4:**
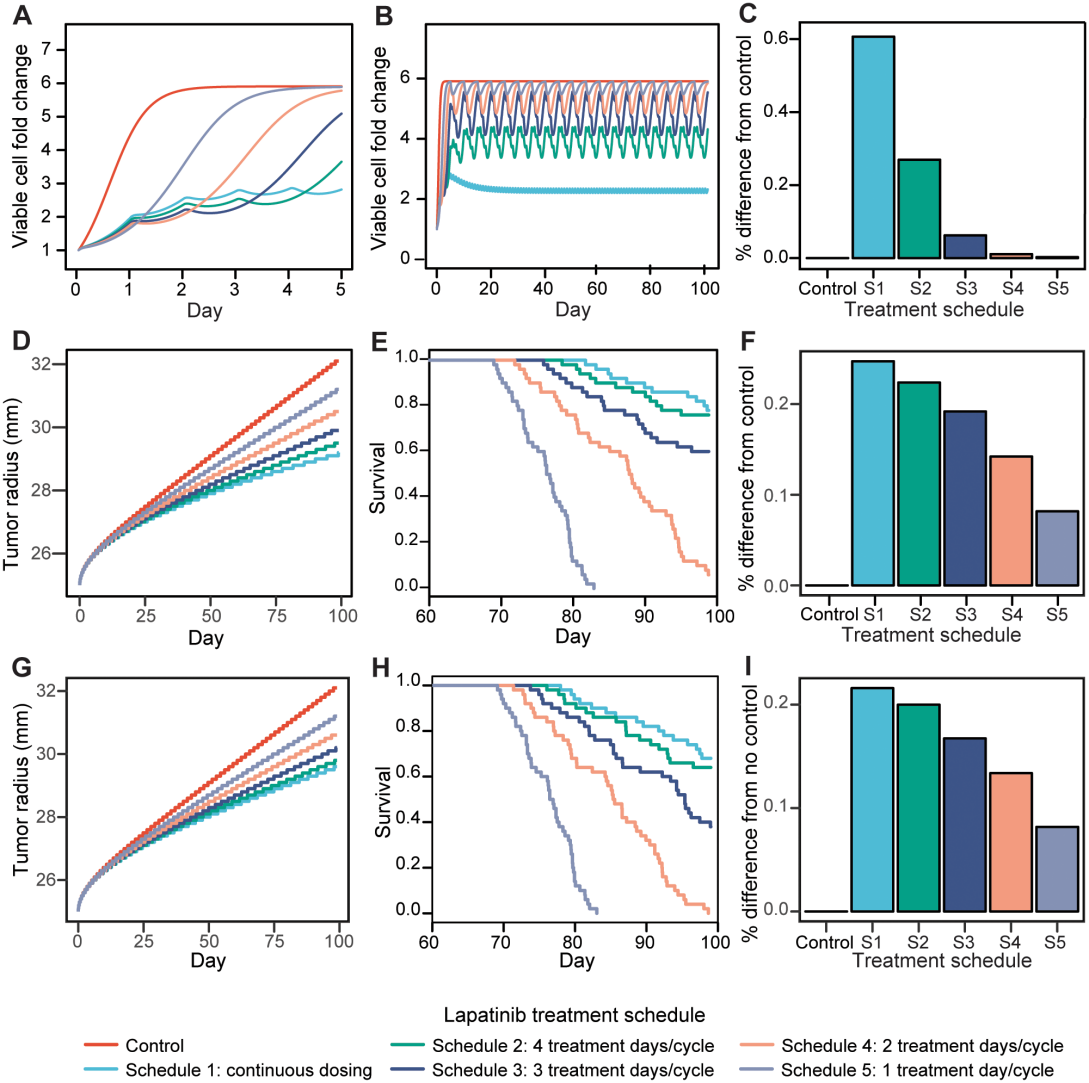
Model-predicted tumor growth trajectories and survival outcomes for five different maximally tolerated dose schedules. A: Predicted short-term tumor growth trajectories for the five MTD treatment schedules (1 treatment cycle) based on the logistic ODE growth model. B: Predicted long-term tumor growth trajectories (20 treatment cycles) for the five MTD treatment schedules based on the logistic ODE model. C: Comparison of the outcomes of the five MTD treatment schedules to the control schedule (no treatment) based on the logistic ODE growth model. The best dosing schedule (smallest tumor volume predicted after 20 treatment cycles) has the highest bar while the control schedule has zero height. D: Predicted long-term tumor growth trajectories (20 treatment cycles) for the five MTD treatment schedules based on the logistic diffusion PDE model. E: Kaplan Meier analysis showing the results of a simulated clinical trial of the five MTD schedules based on the logistic diffusion PDE model. 50 patients were sampled from varying growth rate and PK parameters (see Methods). F: Comparison of the outcomes of the five MTD schedules to the control schedule (no treatment) using the logistic diffusion PDE model. The best dosing schedule (smallest tumor volume predicted after 20 treatment cycles) has the highest bar while the control schedule has zero height. G: Predicted long-term tumor growth trajectories (20 treatment cycles) for the five fixed total dose treatment schedules based on the logistic diffusion PDE model. H: Kaplan Meier analysis showing the results of a simulated clinical trial of the five fixed total dose treatment schedules using the logistic diffusion PDE model. 50 patients were sampled from varying growth rate and PK parameters (see Methods). I: Comparison of the outcomes of the five fixed total dose schedules to the control schedule (no treatment) using the logistic diffusion PDE model. The best dose (smallest tumor volume predicted after 20 treatment cycles) has the highest bar while the control schedule has zero height.

We first investigated the five individual treatment schedules using a logistic ODE growth model (Eq 1), which is the simplest growth model possible under our assumptions. The *in vitro* viable cell trajectories for the five treatment schedules for both short-term response (one 5-day cycle) and long-term response (twenty 5-day cycles) are shown in Fig 4A and 4B. Based on this analysis we observed that none of the five schedules is capable of reducing the total number of viable cells either in the short-term or long-term. In the short-term, all pulsed dosing schedules with large up-front loading doses result in slower viable cell growth than the continuous dose schedule on the initial high dose days. However, once switching to the treatment holidays, viable cell growth in the pulsed schedules outpaces that of the continuous schedule, resulting in a sharp increase in the numbers of viable cells. In the long-term, pulsed schedules result in large fluctuations in the number of viable cells. In contrast, the number of viable cells in the continuous schedule increases smoothly over time. In addition, the number of viable cells in the continuous dosing schedule converges to a limit that is lower than the carrying capacity whereas the maximum numbers of viable cells in the four pulsatile dosing schedules are higher than that in the continuous dosing schedule. Finally, comparing the number of viable cells at the end of treatment to a control schedule (no treatment), we see that the continuous dosing schedule results in a 60% reduction in the total number of viable cells at the end of treatment compared to no treatment, whereas the best pulsatile treatment has less than a 30% reduction (Fig 4C). Although lapatinib fails to arrest further tumor expansion, our data suggest that lapatinib has the ability to reduce overall tumor burden below the carrying capacity under a continuous dosing schedule (schedule 1). Additionally, continuous dosing of lapatinib decreases the number of viable cells at the end of 20 treatment cycles compared to the pulsatile treatment schedules.

#### Optimum dosing strategies using a diffusion PDE growth model

Human GBM is known to be highly diffusive and infiltrative [33, 36, 39–45]. Hence our logistic ODE growth model based on *in vitro* growth may not accurately reproduce some of the important characteristics of human disease as it does not account for tumor cell diffusivity and heterogeneity. In order to better scale our model to human disease, we performed additional analyses using a logistic growth diffusion model (Eq 8). Diffusion models have been widely used to model tumor growth [33, 36, 46–49]; for example, in Swanson et al. [33] the authors showed that they can accurately predict patient survival using a diffusion model for tumor growth, supporting the applicability of such a model for investigating human GBM growth kinetics.

To incorporate the diffusion model into our approach, we used a proliferation rate of *ρ* = 0.387/day and diffusion parameter *D* = 0.03mm^2^/*day*, which represent the patient-derived median proliferation and diffusion rates reported in Murray, 2012 [36]. We let the initial solid and isolated cell tumor radii be 1.5cm and 2.5cm, respectively, where the solid and isolated cell tumor volumes represent tumor cell concentrations of 80% and 16%, respectively, of the maximum cell concentration; 1.5cm was the initial solid tumor radius used in [33], and 2.5cm is in the isolated cell radius range reported in Swanson et al. [33]. Note that we chose 2.5cm rather than the median reported isolated cell radius of 2.84cm to allow simulated tumors sufficient time to grow before reaching a radius of 3cm, which is consistently considered to be the “fatal” radius [33]. Finally, we let the lower threshold for detection of cancer cells on an MRI be 8,000 cells/mm^3^, and the carrying capacity, *κ*, be 10^6^ cells/mm^3^. To model GBM proliferation during treatment, we scaled the proliferation rate by the birth and death rates during treatment estimated from the *in vitro* data (Eq 9). The resulting diffusion model in the presence of treatment is described in Eq 10.

The long-term tumor growth trajectories for the five MTD treatment schedules are shown in Fig 4D. Consistent with our analysis based on the ODE model, we observed that even when using the PDE model, none of the treatment schedules are able to reduce the total tumor volume. However, as before, the continuous dosing schedule again results in slower tumor growth and a reduced tumor volume at the end of treatment compared to the pulsatile dosing strategies (Fig 4F).

Next, we simulated a clinical trial testing the five MTD treatment schedules in a cohort of 50 patients. We varied the GBM growth rates and pharmacokinetic parameters across patients (see Methods) and considered the time of death of a patient to be when their tumor reached 3cm in diameter [33]. The resulting Kaplan-Meier survival distributions are shown in Fig 4E. We observed that patients treated with the continuous dosing schedule (schedule 1) have the best survival outcomes, while patients treated with the one treatment day/cycle schedule (schedule 5) have the worst survival outcomes, which is consistent with the predicted growth rate trajectories in Fig 4D. Moreover, using a log-rank test, we found that the differences between the continuous dosing schedule and schedules 3, 4, and 5 are significant (*p*_*schedule*3_ = 0.0316, *p*_*schedule*4_ = 7.7 × 10^−16^, *p*_*schedule*5_ < 2 × 10^−16^). The difference between continuous dosing and schedule 2 (4 treatment days/cycle) is not significant, which is not surprising because the two schedules are very similar.

We observe that the median survival times predicted by our model (80-90 days for schedules 4 and 5) may underestimate survival times observed in the clinic (12-14 months with agressive treatment). However, our analysis suggests that schedules 4 and 5 are only slightly better than no treatment at all. Thus our results are not unreasonable given that median survival for patients receiving no treatment or only palliative antitumor therapy is 8.8 weeks [50], so we are comfortable using this analysis to compare treatment schedules.

By using the MTD on each treatment day for the individual schedules, the total dose administered during a treatment cycle is different for each schedule because administering more frequent treatments of smaller allotments allows administration of more total dose during a treatment cycle compared to less frequent administrations of large doses. The rationale for this approach is to maximize the total dose for each schedule design while still remaining under the MTD. Alternatively, to investigate whether the differences in continuous vs pulsatile dosing are due to differences in the total amount of drug administered, we also investigated tumor growth during five treatment schedules with a fixed total dose (Table 4). The long-term growth trajectories for the five fixed total dose treatment schedules are shown in Fig 4G, the Kaplan-Meier analyses from the simulated clinical trial using the fixed total dose schedules is shown in Fig 4H, and the comparison of tumor volumes at the end of each treatment are shown in Fig 4I. When implementing this approach, we observed that the ranking of schedules with regard to their treatment efficacies is consistent with our previous results using the maximum tolerated administered dose per day. However, the fixed total dose schedules result in larger tumors at the end of treatment compared to the MTD schedules. Therefore we performed the remainder of our analysis using the MTD schedules listed in Table 3 as our goal is to identify the best overall treatment strategy regardless of the total drug dose administered in one treatment cycle.

**Table 4:** Lapatinib concentrations for fixed total dose treatment schedules.

Schedule	Treatment Days	Oral (mg)	Serum (ng/mL)	In tumor (nM)
1	5	1400	1534	1025
2	4	1750	1661	1074
3	3	2330	1826	1881
4	2	5250	2294	1483
5	1	7000	2460	1590

#### Incorporating intratumor heterogeneity into lapatinib treatment response models

Human tumors contain a large extent of intratumor heterogeneity [51–65] which represents a major cause of drug resistance [51, 61, 62, 66]. To determine the effects of intratumor heterogeneity on lapatinib treatment response, we considered a two cell-type population of tumor cells containing lapatinib-sensitive and -resistant cells. This scenario is represented by the model described in Eq (12). We again investigated the five MTD treatment schedules for this scenario when considering different levels of pre-existing resistance (i.e. 0%, 1%, 5%, and 10% pre-existing resistant cells at the start of treatment).

**Fig 5:**
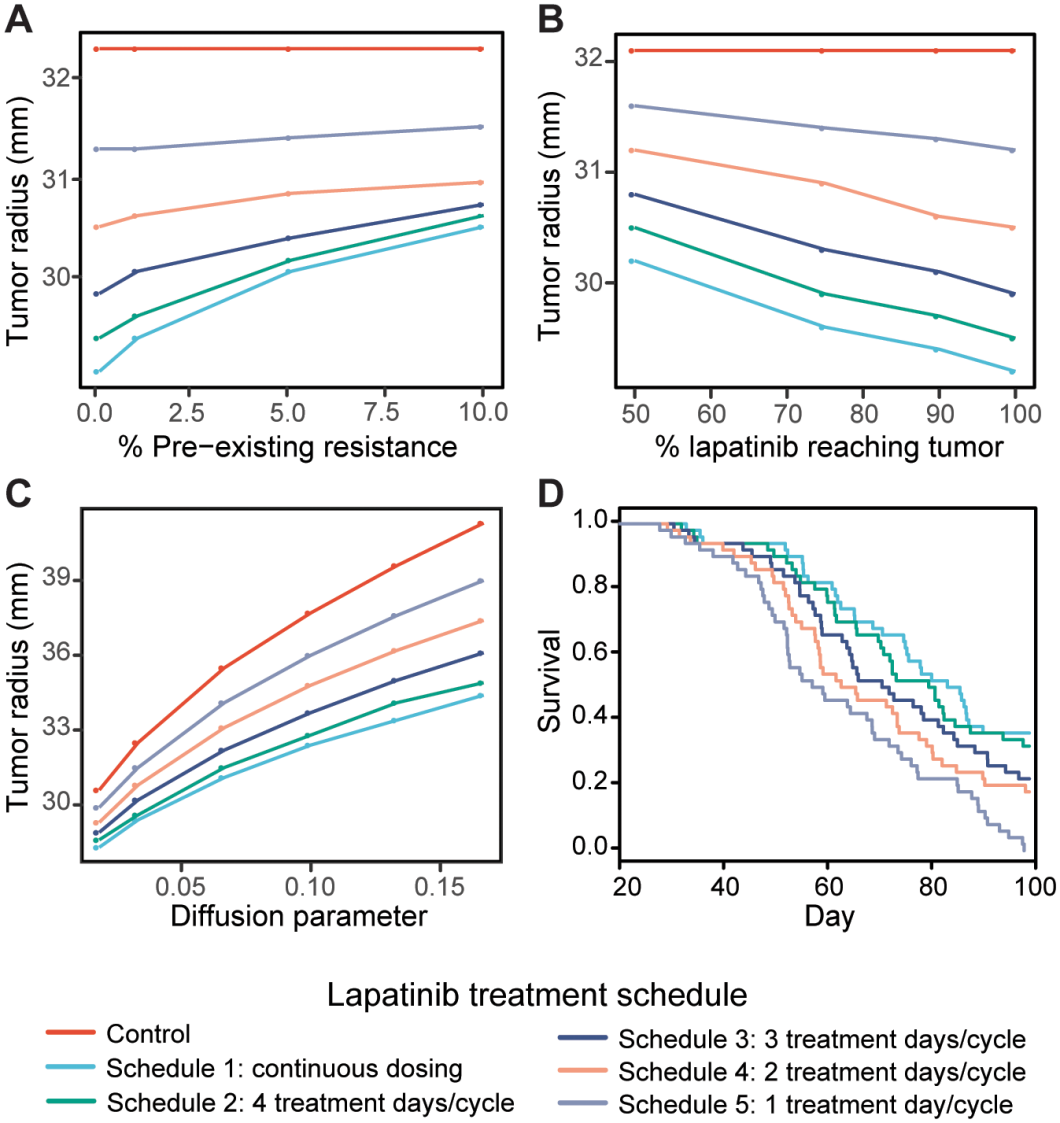
PDE model-predicted effects of intratumor heterogeneity, the blood brain barrier, and differential motility on tumor growth trajectories. A: Comparison of predicted tumor radii at the end of 20 treatment cycles between the five MTD treatment schedules for 0%, 1%, 5%, and 10% pre-existing resistance. B: Comparison of predicted tumor radii at the end of 20 treatment cycles between the five MTD treatment schedules for 50%, 75%, 90%, and 100% of the serum lapatinib concentration entering the tumor. C: Comparison of predicted tumor radii at the end of 20 treatment cycles between the five MTD treatment schedules for diffusion coefficients of 0.0183, 0.033, 0.067, 0.1, 0.133, 0.167 mm^2^/day. D: Kaplan Meier analysis showing the survival outcomes of a simulated clinical trial of 50 patients per schedule with variable diffusion parameter.

The long-term growth trajectories of tumors with varying pre-existing resistance are shown in S2 Fig, and a comparison of tumor volumes at the end of treatment between the five schedules across different levels of pre-existing resistance is shown in Fig 5A. We found that for 0% and 1% pre-existing resistance, continuous dosing leads to better efficacy than any of the pulsatile dosing schedules; however, when there is 5% or 10% pre-existing resistance, the continuous dosing and 4 doses/cycle schedule (schedule 2) perform similarly, though still better than the other pulsatile dosing schedules. These results indicate that the continuous dosing schedule is preferable to pulsatile dosing even in the presence of a resistant cell population, although the difference among the individual treatment schedules becomes smaller at higher levels of pre-existing resistance.

#### Non-uniform treatment delivery to the brain

A major reason for GBM treatment failure is insufficient drug delivery to the brain. The blood brain barrier prevents most systematically-delivered molecules from being transported into the brain [67]. Indeed, the tumor concentrations of lapatinib in the patient cohort from Vivanco et al. [32] vary considerably, ranging from 70-3826 nM, as do the plasma/tumor ratios, and the blood brain barrier may be one reason causing such variable lapatinib concentration reaching the tumor.

To establish if the blood brain barrier affects lapatinib treatment response, we made the assumption that the blood brain barrier causes less drug to enter the tumor leading to a decreased drug effect. We adapted our diffusion model to account for reduced drug delivery to the brain by introducing a factor *Q* in the proliferation rate function (Eq 13). We use this model to assess how much a 10%, 25%, and 50% decrease in the lapatinib concentration reaching the tumor from the serum affects our simulated treatment outcomes. The growth trajectories resulting from different amounts of lapatinib reaching the tumor for each treatment schedule are shown in S3 Fig. We also compared tumor volumes at the end of treatment between treatment schedules across different levels of lapatinib reaching the tumor (Fig 5C). As expected, we observed that decreasing the amount of lapatinib that crosses the blood brain barrier decreases the effect of treatment. However, the decreased effect of treatment is similar across all treatment schedules; hence continuous dosing still remains the best treatment option for reducing long-term tumor growth, even when there is reduced availability of lapatinib in the brain.

#### The effects of motility on lapatinib treatment response

Tumor cell motility has been described as a source of variability in treatment responses among a patient cohort and measures of motility can be predictive of treatment response [36]. In order to asses the effects of tumor cell motility on treatment outcomes, we simulated tumor growth using Eq (8) for varying values of the diffusion parameter, *D*, estimated from patient data (ref). For our analyses we utilized *D* equal to 0.01833, 0.0333, 0.0667, 1.333, and 1.667 mm^2^/day, where 0.01833 mm^2^/day is the lower limit and 1.667 is above the upper limit reported in Murray, 2012 [36]. Long-term tumor growth for each diffusion parameter is shown in S4 Fig. We additionally compared tumor volumes at the end of treatment between schedules across the diffusion parameters (Fig 5C). Although we found, as expected, that tumors with higher motility grow faster during all treatment schedules, the continuous dosing schedule performs better than all pulsatile dosing strategies across all diffusion parameters tested. We then investigated whether the relationship between motility and tumor volume (in mm^3^) at the end of treatment depends on the treatment schedule using linear regression with an interaction term between the diffusion parameter and the schedule (Eq 17). We found that schedules 3, 4, and 5 are significantly more affected by increased motility than schedule 1 (Table 5), indicating that the effectiveness of continuous dosing is less comromised by increased cell motility than that of a pulsatile schedule with only 1, 2, or 3 treatment days per cycle. This observation suggests that a continuous dosing strategy is more effective than pulsatile dosing even with a large extent of cell motility, consistent with our previous results.

**Table 5:** Coefficient estimates for the effects of GBM cell motility on tumor growth.

Parameter	Estimate	Standard Error	p-value
*ν*_0_	89,410	3,021	<2 × 10^−16^
*ν*_*D*_	501,579	29,853	2.92 × 10^−13^
*ν*_*schedule*2_	1739	4272	0.6883
*ν*_*schedule*3_	4162	4272	0.3417
*ν*_*schedule*4_	7304	4272	0.1028
*ν*_*schedule*5_	10982	4272	0.0183
*ν*_*int*, *schedule*2_	43,666	42219	0.3134
*ν*_*int*, *schedule*3_	138,696	42219	0.0037
*ν*_*int*, *schedule*4_	258,064	42219	5.66 × 10^−6^
*ν*_*int*, *schedule*5_	411,360	42219	4.89 × 10^−9^

Tumor motility heavily depends on the location of the tumor in the brain, as malignant glial cells diffuse more quickly in white matter than gray matter [68], [69]. Thus, tumor motility can be highly variable between patients, depending on where their tumors are located. To further investigate how variability of motility across a patient cohort affects treatment outcomes and survival, we simulated a clinical trial of the five MTD treatment schedules with a varying diffusion parameter in addition to varying cell dynamics and pharmacokinetics parameters (see Methods). The resulting Kaplan Meier analysis is shown in Fig 5D. We observed that patients treated with a continuous dosing schedule have the longest survival, whereas patients treated with schedule 5 have the worst survival outcomes, consistent with our previous results. Using a log-rank test, the difference between the continuous dosing schedule and schedules 4 and 5 are significant (*p*_*schedule*4_ = 0.0104, *p*_*schedule*5_ = 1.33 × 10^−6^).

#### Incorporating the Go-or-Grow mechanism into our mathematical framework

Glioblastoma is known to be highly hypoxic [34, 70], and tumor cell invasion is one of many different rescue mechanisms that allow tumors to survive hypoxic conditions [34, 71]. According to the Go-or-Grow hypothesis, in low oxygen conditions, tumor cells undergo a phenotypic switch from proliferative to invasive/migratory phenotypes [72]. Multiple mathematical models have been developed to describe the effects of this phenomenon on tumor growth [35, 73–76]. We adapted Model II described in [35] to investigate the five laptinib treatment schedules under the assumption of the Go-or-Grow model. Our model is decribed in Eqs 14–16 and parameterized using experimentally derived values, as in [35]. We used vascular density $v=\frac{1}{2}$; oxygen concentration in normal tissue *σ*_0_ = 2.068 × 10^−9^ mol mm^−3^; proliferative to migratory switching parameter λ_1_ = 4.134 nmol mm^−3^ (twice the normal tissue concentration); migratory to proliferative switching paramter λ_2_ = 0.5, 1, and 2; oxygen diffusion rate *D*_*σ*_ = 1.51 × 10^2^ mm^2^ day^−1^; oxygen supply rate *h*_1_ = 3.37 × 10^−1^ day^−1^; and oxygen consumption rate *h*_2_ = 10^−2^ mm cell^−1^ day^−1^ (in the range of values reported in [35]). When implementing this model, we found that continuous dosing performs better than all pulsatile dosing schedules (S5 Fig), consistent with our results above.

## Discussion

Here we have presented a computational analysis platform of human GBM growth and treatment response, parameterized using *in vitro* tumor cell data measured based on the SF268 GBM cell line during lapatinib exposure. Our data demonstrate that lapatinib concentrations are negatively correlated with cell birth rates, but positively correlated with death rates. However, the necessary lapatinib concentration needed to arrest further tumor expansion is calculated to be 2,180nM *in vitro*, a concentration that cannot be reached in GBM patients based on current toxicity limits and oral absorption profiles. To achieve this concentration, our absorption model predicts that an oral dose of 34,000mg is required—5 times greater than the highest dose ever tested. The therapeutic potential seems to be restricted by the transport of lapatinib from the gastrointestinal tract to tumor sites. Due to the logarithmic relationship between oral intake and serum concentration, a higher than tolerated dose is required to achieve tumor reduction. Other methods of delivery, such as intravenous injection, may need to be considered to bypass the current delivery problem. This result agrees with the conclusions from early clinical trials that lapatinib failed to reduce tumor sizes [15], [16]. Despite these negative results, our model suggests that continuous lapatinib dosing does have positive effects. Continuous dosing was able to reduce long-term tumor burden significantly more than pulsatile treatment schedules. These findings were consistent with incorporating intratumor heterogeneity due to the presence of drug-resistance cells, variable penetration of the blood brain barrier leading to a reduction of lapatinib concentration in the tumor, and tumor cell motility.

Optimization of dosing strategies for cancer treatments is a complex problem. Our mathematical model provides a quantitative relationship bridging *in vitro* tumor dynamics and *in vivo* lapatinib dose and schedule, taking into account the blood brain barrier, GBM motility, heterogeneity, and phenotypic changes that occur in hypoxic microenvironments. Our approach allows for the systematic comparison between different treatment strategies and their effects on tumor growth. Although none of the tolerable doses and schedules evaluated were found capable of arresting tumor expansion, our approach predicts that continuous treatment within these parameters is likely to be more successful at slowing down tumor growth. Further optimization of effective dosing strategies using our computational platform will benefit from additional dose-limiting toxicity studies that incorporate multiple pulsatile schedules.

## Supporting information

S1 Fig Birth, death and clearance rates for different concentrations of lapatinib. A-C: There is no obvious association between birth and death rates with lapatinib concentrations when clearance rates are allowed to vary. D-F: Constraining the clearance rates to be zero, birth rates decrease with increasing lapatinib concentrations and death rates increase lapatinib concentrations. The solid black lines show the relationships between birth and death rates and lapatinib concentration assuming an exponential function. The exponential function is selected to ensure that birth and death rates are always positive for all concentrations of lapatinib.
(TIF)

S2 Fig Long-term growth trajectories for variable pre-existing resistance. A-D: Predicted long-term growth trajectories (20 treatment cycles) for the five MTD schedules with 0%, 1%, 5%, and 10% pre-existing resistance based on the logistic diffusion PDE model.
(TIF)

S3 Fig Long-term growth trajectories for reduced lapatinib penetration of the blood brain barrier. A-D: Predicted long-term growth trajectories (20 treatment cycles) for the five MTD schedules with 50%, 75%, 90%, and 100% of serum lapatinib concentrations penetrating the blood brain barrier and entering into the tumor based on the logistic diffusion PDE model.
(TIF)

S4 Fig Long-term growth trajectories for variable diffusion parameter. A-D: Predicted long-term growth trajectories (20 treatment cycles) for the five MTD schedules with diffusion parameters equal to 0.0183, 0.033, 0.067, 0.1, 0.133, 0.167 mm^2^/day based on the logistic diffusion PDE model.
(TIF)

S5 Fig Long-term growth trajectories under the Go-or-Grow mechanism. A-C: Predicted long-term growth trajectories (20 treatment cycles) for the control and five MTD schedules with migratory to proliferative switching parameter λ_2_ = 0.5, 1, 2.
(TIF)
