# Supplementary figures and images for: Mathematical modeling identifies optimum lapatinib dosing schedules for the treatment of glioblastoma patients
Source: PLoS Comput Biol. 2018 Jan 2;14(1):e1005924. doi: 10.1371/journal.pcbi.1005924 (PMC5766249; doi:10.1371/journal.pcbi.1005924)

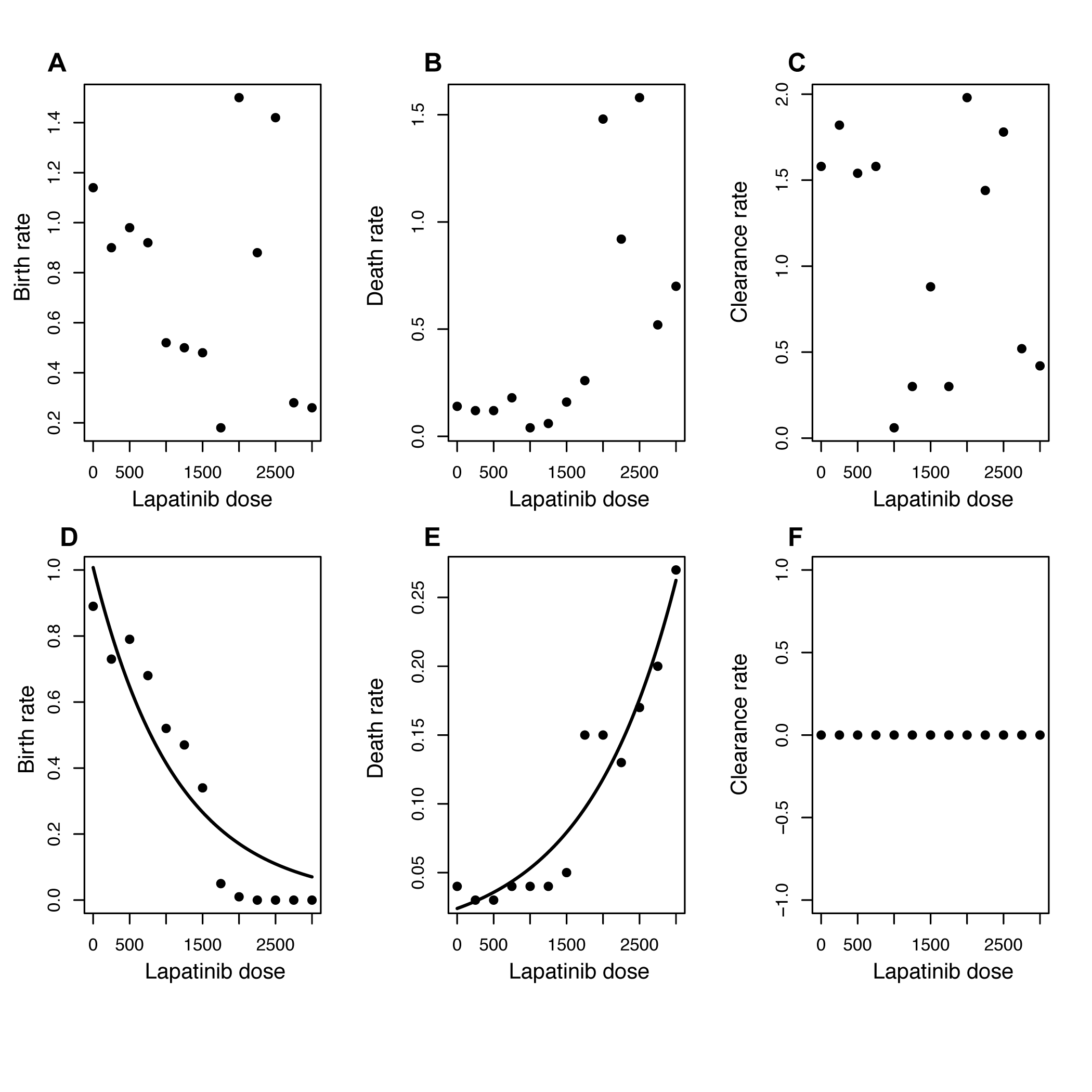

Supplement: S1 Fig — A-C: There is no obvious association between birth and death rates with lapatinib concentrations when clearance rates are allowed to vary. D-F: Constraining the clearance rates to be zero, birth rates decrease with increasing lapatinib concentrations and death rates increase lapatinib concentrations. The solid black lines show the relationships between birth and death rates and lapatinib concentration assuming an exponential function. The exponential function is selected to ensure that birth and death rates are always positive for all concentrations of lapatinib. (TIF) [file pcbi.1005924.s001.tif]

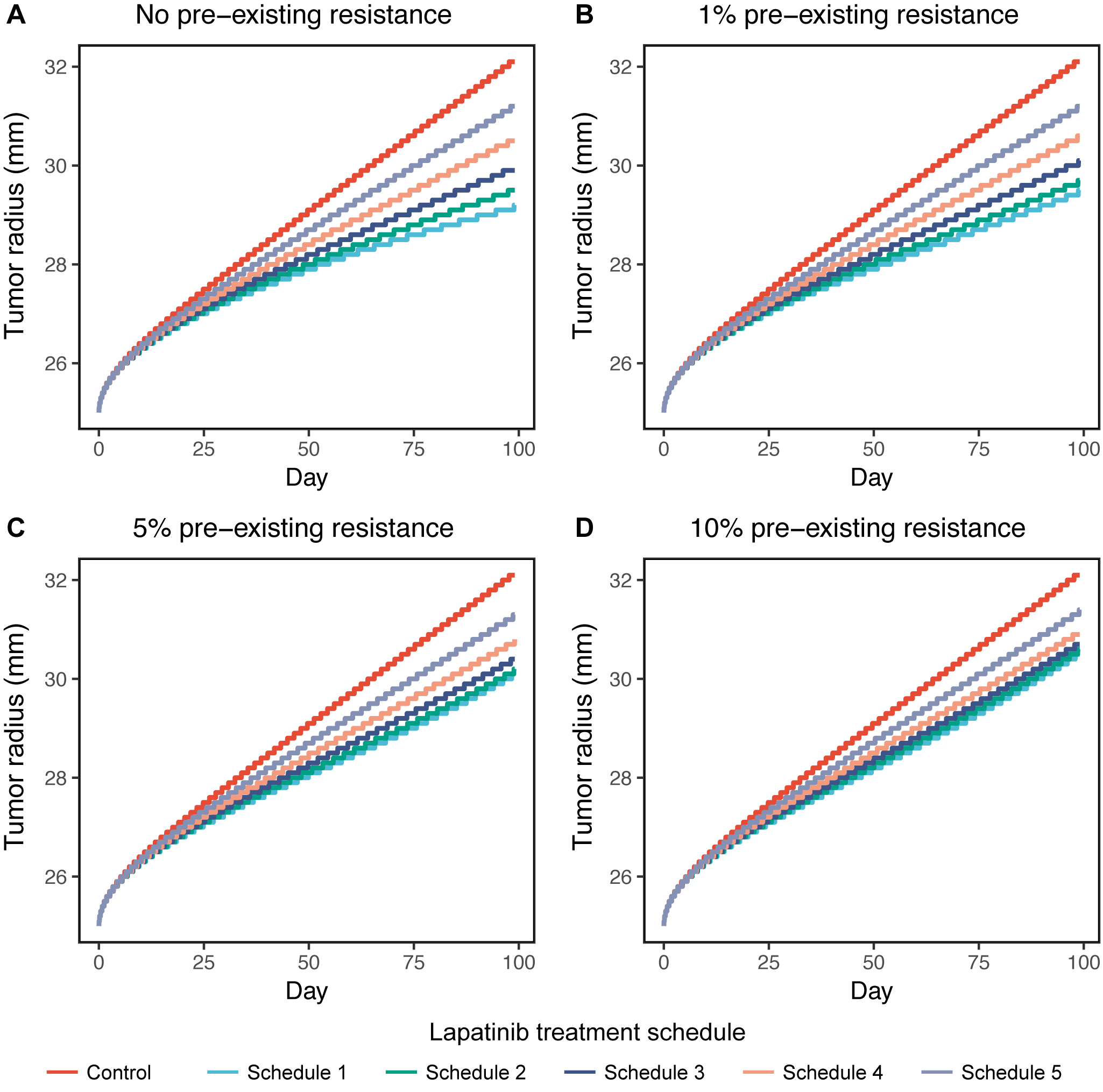

Supplement: S2 Fig — A-D: Predicted long-term growth trajectories (20 treatment cycles) for the five MTD schedules with 0%, 1%, 5%, and 10% pre-existing resistance based on the logistic diffusion PDE model. (TIF) [file pcbi.1005924.s002.tif]

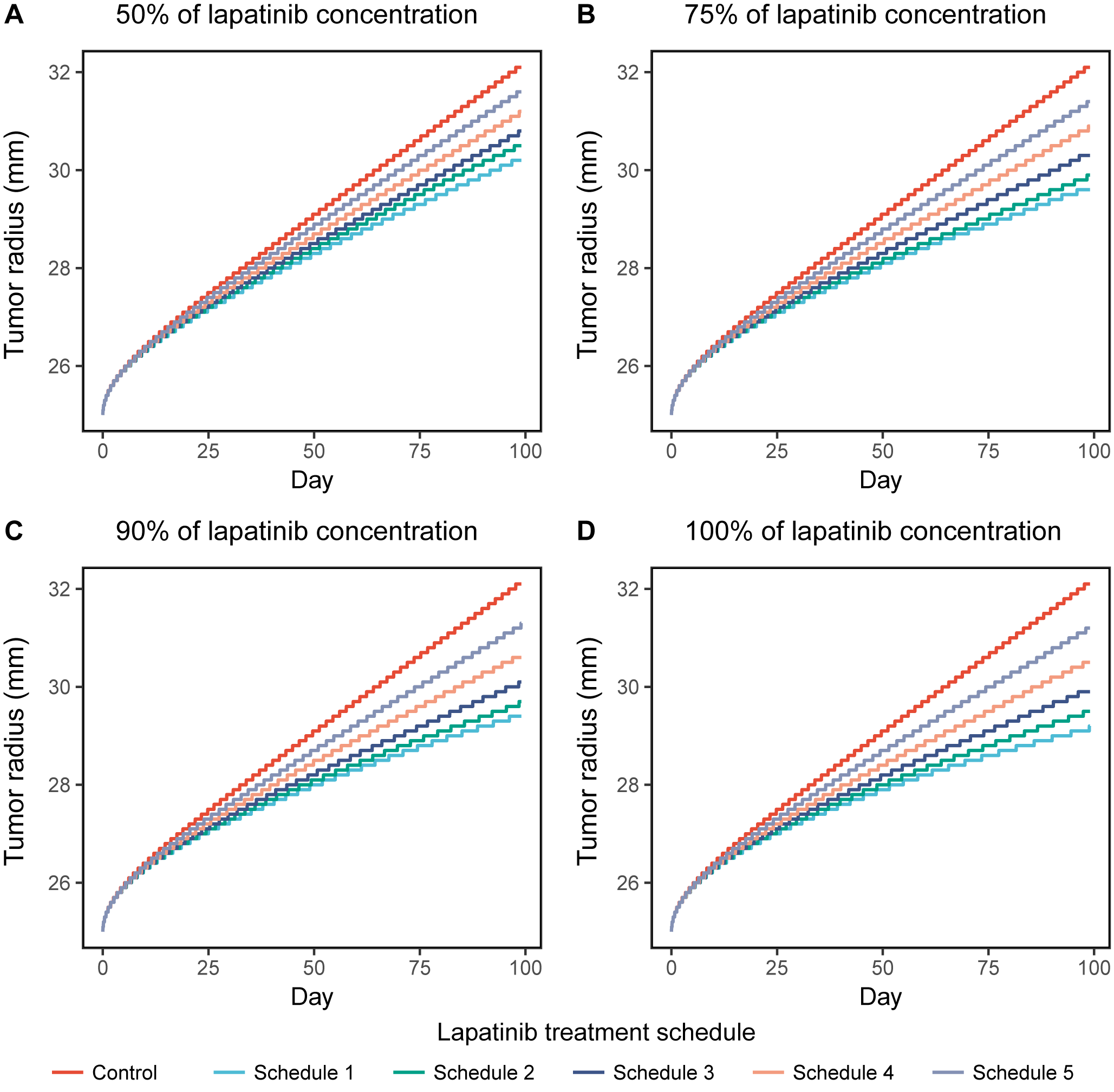

Supplement: S3 Fig — A-D: Predicted long-term growth trajectories (20 treatment cycles) for the five MTD schedules with 50%, 75%, 90%, and 100% of serum lapatinib concentrations penetrating the blood brain barrier and entering into the tumor based on the logistic diffusion PDE model. (TIF) [file pcbi.1005924.s003.tif]

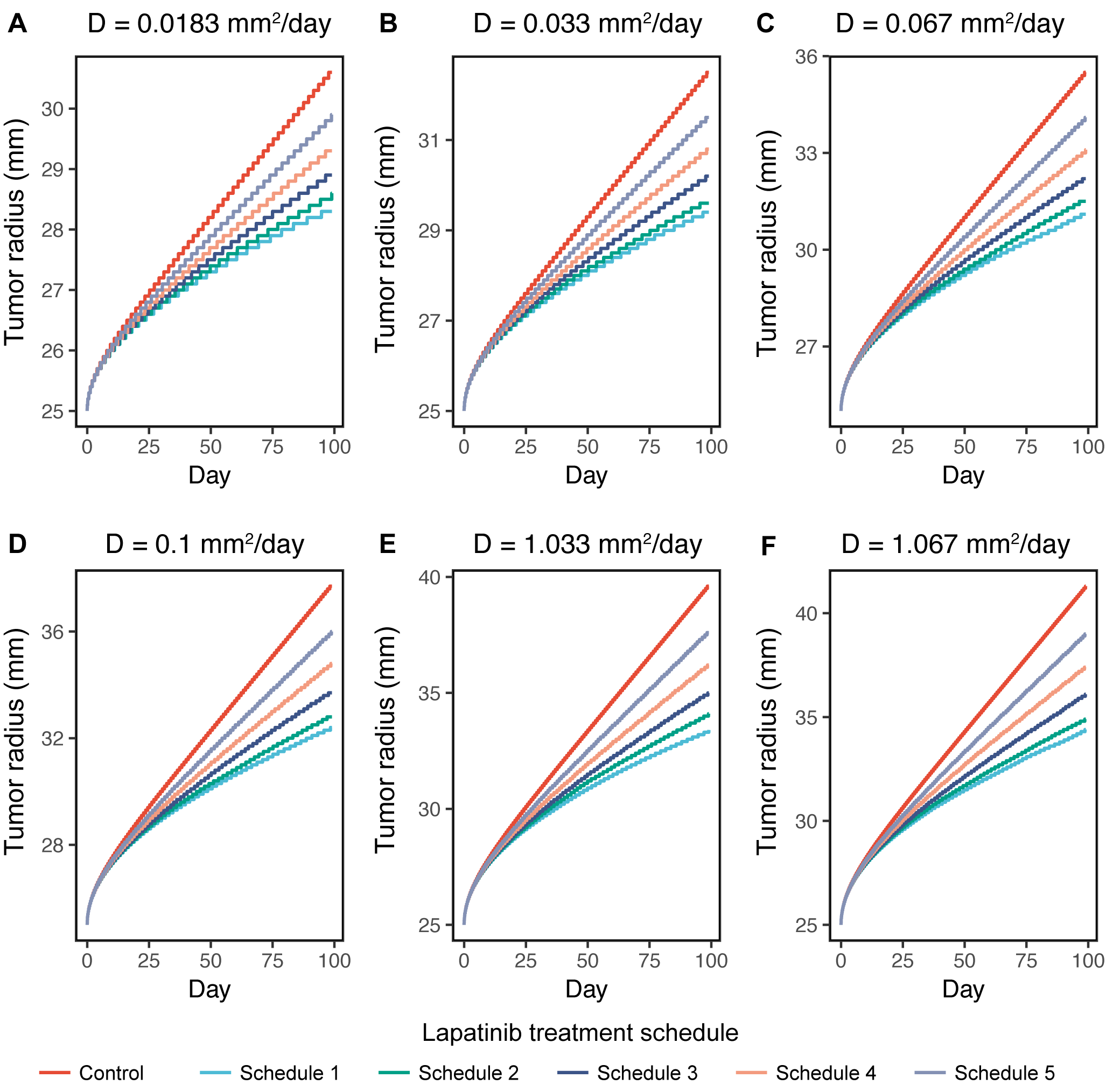

Supplement: S4 Fig — A-D: Predicted long-term growth trajectories (20 treatment cycles) for the five MTD schedules with diffusion parameters equal to 0.0183, 0.033, 0.067, 0.1, 0.133, 0.167 mm2/day based on the logistic diffusion PDE model. (TIF) [file pcbi.1005924.s004.tif]

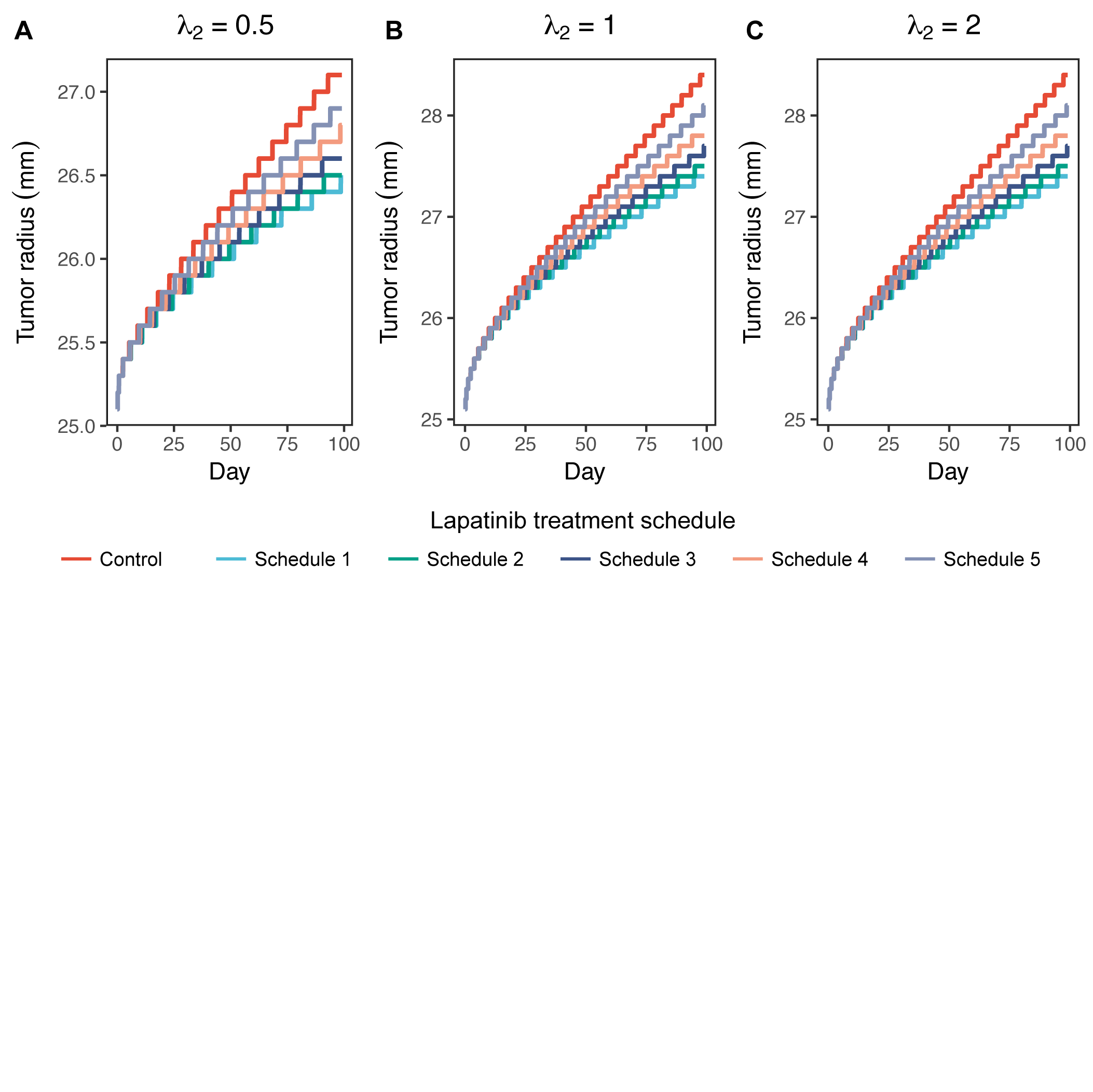

Supplement: S5 Fig — A-C: Predicted long-term growth trajectories (20 treatment cycles) for the control and five MTD schedules with migratory to proliferative switching parameter λ2 = 0.5, 1, 2. (TIF) [file pcbi.1005924.s005.tif]
